# EVOO Polyphenols Exert Anti-Inflammatory Effects on the Microglia Cell through TREM2 Signaling Pathway

**DOI:** 10.3390/ph16070933

**Published:** 2023-06-27

**Authors:** Manuela Leri, Marzia Vasarri, Federica Carnemolla, Francesco Oriente, Serena Cabaro, Maria Stio, Donatella Degl’Innocenti, Massimo Stefani, Monica Bucciantini

**Affiliations:** 1 Dipartimento di Scienze Biomediche Sperimentali e Cliniche “Mario Serio”, Università degli Studi di Firenze, 50134 Firenze, Italy; manuela.leri@unifi.it (M.L.); marzia.vasarri@unifi.it (M.V.); federicacarnemolla95@hotmail.it (F.C.); maria.stio@unifi.it (M.S.); donatella.deglinnocenti@unifi.it (D.D.); massimo.stefani@unifi.it (M.S.); 2 Dipartimento di Scienze Mediche Traslazionali, Università di Napoli Federico II, 80138 Napoli, Italy; foriente@unina.it (F.O.); serena.cabaro@unina.it (S.C.)

**Keywords:** neuroinflammation, TREM2, polyphenols, microglia polarization

## Abstract

In Alzheimer’s disease (AD), microglia, brain resident immune cells, become chronically inflammatory and neurotoxic. In recent years, neuroinflammation has attracted particular interest in the scientific community. The genetic variants of molecules associated with ‘‘microgliopathies’’, including the triggering receptor expressed in myeloid cells-2 (TREM2), result in increased risk of developing AD and cognitive decline. We performed a set of in vitro assays using human neuronal (SH-SY5Y) and microglial (BV2 and C13NJ) cell models. Cells were differentially treated with extra virgin olive oil (EVOO) polyphenols, oleuropein aglycone (OleA) and hydroxytyrosol (HT) before adding LPS. We evaluated the protective effects of these EVOO products by a set of biochemical and cell biology assays, including ELISA, MTT, ROS detection, Western blotting and immunofluorescence. Our results provide an integrated understanding of the neuroprotection exerted by polyphenols in terms of: (i) reduction of pro-inflammatory cytokines release (IL-6, IL-8, IP-10 and RANTES); (ii) activation of the TREM2-dependent anti-inflammatory pathway; (iii) enhancement of protective microglial activity favoring the M2 polarization phenotype. Such findings provide new and important insights into the mechanisms by which the dietary olive polyphenols exert beneficial properties against neuroinflammation and neuronal impairment.

## 1. Introduction

Inflammation plays a pivotal function in neurodegenerative disorders, such as Alzheimer’s disease (AD). Therefore, neuroinflammation is become an important pathological hallmark of the progression and development of these diseases. Microglial cells are the “macrophages” of the central nervous system (CNS) charged with the initiation of the innate immune response to counteract CNS injuries [1]. They, together with astrocytes, contribute in the maintenance of brain homeostasis, and their activation in response to an insult is followed by the release of pro-inflammatory cytokines and reactive oxygen species (ROS) in order to limit injury and initiate repair processes. The loss of the homeostatic function of microglia, during aging or following the presence of triggering factors, results in a persistent inflammatory reaction that causes functional and structural changes, eventually leading to neuronal degeneration [2]. Based on their morphological and functional state, microglia are classified into different phenotypes and functions, i.e., resting (ramified) and activated/phagocytic (ameboid) microglia [3], that undergo modifications during aging and neurodegeneration [4,5,6]. In other settings, activated microglia, under different environmental conditions, are described as susceptible to polarization in terms of pro- or anti-inflammatory states, releasing neurochemicals with neuroprotective or neurotoxic effects, referred to as M1 or M2 phenotypes, respectively, depending on the conditions and the stimulus involved [7,8]. In AD and in several other neurodegenerative diseases, the microglia phenotype also changes to the chronically inflammatory and neurotoxic pathological one (MGnD) [9].

Pathologically, AD is marked by the buildup of extracellular senile plaques mainly containing the polymerized 42 kDa amyloid-β peptide (Aβ_42_) and of intracellular neuronal fibrillar tangles (NTFs) resulting from the polymerization of the hyperphosphorylated tau (p-tau) protein, as well as by reactive gliosis, including microgliosis [10,11]. In recent years, neuroinflammation has attracted particular interest; increased risk of developing AD and cognitive decline is associated with genetic variants of molecules associated with ‘‘microgliopathies’’, including the 12 kDa DNAX activating protein (DAP12) and the triggering receptor expressed in myeloid cells-2 (TREM2) in Nasu-Hakola disease (NHD) [10,11,12].

TREM2 is primarily expressed by microglia where it controls and directs microglial functions in response to the presence of AD-specific pathological markers such as Aβ_42_ plaques and NTFs [11]. Physiologically, TREM2 supports microglial cell survival by stimulating their proliferation and by inhibiting apoptosis, autophagy and, possibly, pyroptosis [13]. In microglia, TREM2 also supports a higher metabolic rate while dysfunctional TREM2 hinders brain metabolism [14,15]. The importance of TREM2 for the healthy state of neuronal cells first resulted from genetic studies showing that heterozygous rare variants of TREM2 (R47H, R62H and H157Y) are linked to an increased risk of developing late-onset AD (LOAD) [16] in European, African, American and Asian populations [17]. A growing body of evidence provides new insight into the multifaceted role of TREM2 in regulating the pathological effects provided, in animal AD models and in AD patients, by the extracellular Aβ peptide fibrils [18], the hyperphosphorylation and aggregation of the tau protein [11,19], microgliosis and the ensuing inflammatory reaction [20,21,22,23,24]. Interestingly, it is worth noting that TREM2 can be cleaved by the γ-secretase, which is also involved in the production of the family of Aβ peptides from the Alzheimer’s precursor protein (APP) in the amyloidogenic pathway [25]. Many potential AD-associated TREM2 ligands, including Aβ_42_, ApoE and anionic phospholipids, have been proposed. Recently, Krasemann et al. identified the TREM2-APOE pathway as a considerable regulator of the switch of the microglia phenotype in neurodegenerative diseases, which may serve as a target to restore microglia homeostasis [9]. Moreover, TREM2 was found to interact with high affinity with soluble toxic Aβ_42_ oligomers [26]; such an interaction resulted in TREM2 stimulation that, in turn, was involved in microglial response to pathogens and other triggers of cell injury. For example, numerous cellular modifications depend on TREM2 activation; the latter include Aβ-induced microglial depolarization, K^+^ inward current induction, cytokine expression and secretion, migration, proliferation, apoptosis and morphological changes [27]. Overall, these data have supplied key insights into the function of TREM2 in Aβ pathogenesis, where it is powerfully involved in the response of the microglia to the presence of amyloid plaques [28].

In consideration of the lack of success of the clinical trials targeting brain accumulation of Aβ_42_ and hyperphosphorylated tau, a better knowledge of the role of TREM2 in the microglia functional states is expected to advance our understanding of the TREM2–AD relation. This can be of importance considering that the loss of TREM2 function increases amyloid seeding in the early stages of AD, whereas, in the advanced stages, increased TREM2 activity correlates with fast plaque progression [29]. This curious phenomenon could be the result of stimulation of microglia by TREM2 to produce ApoE, which, in turn, stimulates plaque formation [30].

The TREM2–AD relation could be of importance in the search for novel therapeutic strategies for the medical therapy of AD and other neurodegenerative diseases, for which current therapies merely provide relief to acute symptoms, while brain neurodegeneration proceeds exponentially. By promoting neurotoxic environments associated with neurodegeneration, both oxidative stress and neuroinflammation precede the appearance of the functional symptoms in neurodegenerative disorders. In this context, plant polyphenols have the potential to serve as a new tool to be used for prevention and, possibly, the slowing down of neurodegenerative disorders; in fact, they have been reported to restrict the activation of oxidative networks and the inflammatory reaction after cell exposure to environmental stressors.

In this study, we evaluated the anti-neuroinflammatory properties of the main polyphenols in extra virgin olive oil (EVOO), oleuropein aglycone (OleA), and its main metabolite, hydroxytyrosol (HT), in neuronal and glial cultured cells; accordingly, we discussed the possible use of these molecules to treat, delay or ameliorate the onset and progression of chronic neurodegeneration. Recent data indicate that OleA and HT exhibit pleiotropic neuroprotective effects, including the anti-aggregation, anti-inflammatory and antioxidant power [31,32]. Moreover, OleA arouses cell defenses against plaque-induced neurodegeneration, activates autophagy, a function commonly compromised in neurodegenerative contexts, together with synaptogenesis both in cultured cells [33] and in an OleA-fed animal model of plaque deposition [34,35]. HT also shows anti-inflammatory and antioxidant power both in cultured cells, where it reduces inflammatory markers, cyclooxygenase-2 (COX2) and Tumor Necrosis Factor alpha (TNF-α), and in vivo, in a mouse model of systemic inflammation [36]. Accordingly, it is reasonable to assume that the biological activity of OleA and HT may interfere with the meshwork of interactions and interdependencies between microglia and neurons. On the basis of microglia involvement in AD pathogenesis, finding the right therapy, capable of activating the microglia only at the right moment, is not an easy task, yet it is an exciting challenge.

Our study will contribute to outlining a coherent picture of the complex biological activity underlying neuroprotection by OleA and HT and hence, the conceptual substrate needed to identify new therapeutic targets and strategies against AD. It will also increase the knowledge of puzzling processes, such as the neurodegenerative shift of neuroinflammation, that characterize this pathology.

## 2. Results

### 2.1. OleA and HT Reduce Oxidative Stress and Mitochondrial Damage in BV2 Cells Exposed to LPS

**Figure 1:**
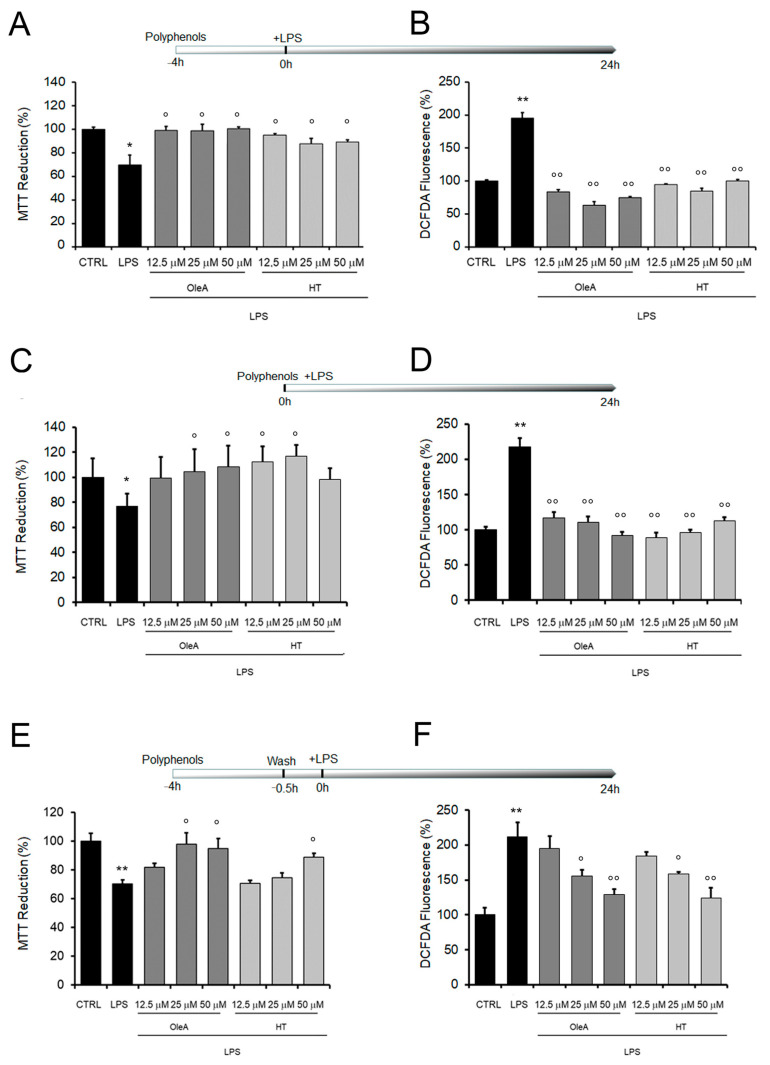
Mitochondrial damage and oxidative stress induced by cell exposure to LPS are reduced by OleA and HT. BV2 cells starved for 16 h were exposed to different concentrations of OleA or HT (12.5, 25, 50 μM) for 4 h and then challenged with LPS (1.0 μg/mL) for 24 h (**A**,**B**); co-treated with different concentrations of OleA or HT and LPS (1.0 μg/μL) for 24 h (**C**,**D**); exposed to different concentrations of OleA or HT (12.5, 25, 50 μM) for 4 h, then washed 2 times with PBS, 10 min each time before LPS (1.0 μg/μL) supplementation to the culture media and incubation for 24 h (**E**,**F**). (**A**,**C**,**E**) Cytotoxicity, as assessed by the MTT assay. (**B**,**D**,**F**) ROS levels, as measured by the DCFDA probe. All experiments were reported as the mean ± SE of three independent experiments in respect to untreated cells (CTRL). Statistics: *: *p* < 0.05; **: *p* < 0.01 vs. untreated cells (CTRL). °: *p* < 0.05; °°: *p* < 0.01 vs. LPS treated.

We started our study by analyzing the cellular stress induced by exposing cultured BV2 cells to increasing concentrations of LPS (1.0 to 20 μg/mL). LPS-induced BV2 microglia cells are a widely used model to investigate the inflammatory response, since LPS, as an endotoxin, can induce oxidative stress and inflammatory stimuli, with the release and accumulation of inflammatory molecules from exposed cells. We first assessed mitochondrial sufferance and the intracellular ROS production in LPS-exposed BV2 cells by using the MTT assay and the DCFDA probe, respectively. We found that LPS induced a dose-dependent cell sufferance and ROS production (Supplementary Figure S1); among the tested LPS doses, BV2 treatment with 1.0 mg/mL LPS for 24 h was chosen as the optimized concentration in this study; the latter dose induced a decrease of cell viability up to 31 ± 1.72% and an increase of 195 ± 8.26% of oxidative stress respect to untreated cells. Cells exposed only to either OleA or HT did not exhibit a significant difference in cell distress and ROS levels compared to untreated cells (Supplementary Figure S1). Then, we sought to assess the impact of OleA and HT on the toxicity induced by LPS to BV2 cells. The cells were exposed to OleA or HT at concentrations ranging from 12.5 to 50 μM for 4 h before adding LPS to the culture media for further 24 h. Cells pre-exposed to OleA before LPS supplementation displayed viability and ROS levels similar to those measured in control cells at all polyphenol concentrations used (100 ± 5.4%) (Figure 1A). Cells pre-exposed to HT displayed a viability of 87 ± 5.16% at the lower dose and of 89 ± 9.3% at the higher dose used (Figure 1B). Finally, the simultaneous co-treatment for 24 h of the cells with LPS (1.0 μg/mL) and with either polyphenol OleA and HT induced a complete recovery of viability and decrease of ROS levels, particularly when the cells were exposed to OleA at all three concentrations used (Figure 1C,D).

Once we had assessed the protection of either polyphenol against LPS cytotoxicity, we evaluated the ability of OleA and HT to adhere at the cell membrane and the ability of the cells to retain polyphenol molecules. To do this, we washed the cells two times with PBS after exposure to either polyphenol and before LPS supplementation to the culture media. We found that the cells treated with either polyphenol were still significantly protected from LPS stress at the highest concentrations of polyphenol used (25 and 50 μM for OleA, 50 μM for HT), as reported in Figure 1E,F. The maintenance of cell protection after washing suggests the possibility that both OleA and HT are able to interact with the BV2 cell membrane, even though polyphenol internalization inside the exposed cells cannot be excluded.

### 2.2. LPS Induces a Decrease in TREM2 Levels Which Is Prevented by Cell Exposure to Either Polyphenol

**Figure 2:**
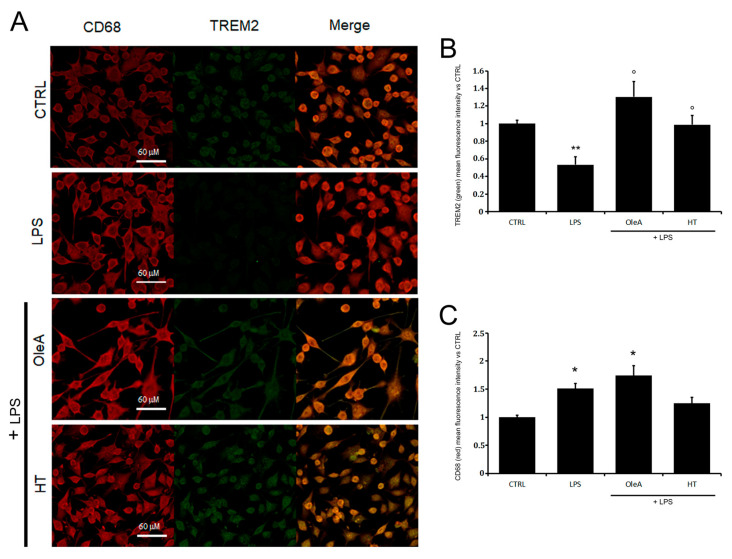
EVOO polyphenols reduce TREM2 imbalance. (**A**) Representative images and (**B**,**C**) semi-quantitative analysis of BV2 cells starved for 16 h (CTRL, untreated cells) and then exposed for 24 h to LPS (1.0 μg/mL) in the absence or in the presence of a pre-treatment for 4 h with 25 μM OleA or with 25 μM HT. In the images, the red fluorescence corresponds to the cell membrane labeled with mouse primary antibody anti-CD68 and secondary anti-mouse 568-conjugated; green fluorescence corresponds to TREM2 labeled with the specific primary anti-TREM2 rabbit antibody and the secondary anti-rabbit Alexa 488-conjugated antibody. Scale bar, 60 μm. The two channels are shown separately and in merge mode. The reported data are representative of three independent experiments. (**B**) Mean values of the green fluorescence (TREM2) signals reported vs. untreated cells (CTRL). (**C**) Mean values of the red fluorescence (CD68) signals reported vs. untreated cells (CTRL). Semi-quantitative analysis was performed by ImageJ software as integrated intensity of at least 3 images for each of 3 independent experiments. Statistics: *: *p*< 0.05; **: *p* < 0.01 vs. CTRL; °: *p* < 0.05 vs. LPS.

Recent data indicate that TREM2 expression in microglia and subsequent anti-inflammatory signaling is suppressed by LPS-induced pro-inflammatory stimulus, Therefore, to evaluate a possible effect of OleA or HT during the acute inflammatory response, we assessed TREM2 levels after exposure of BV2 cells to LPS in the absence or in the presence of either polyphenol. Cells starvation and cells exposure to each polyphenol, either alone (12.5, 25, 50 μM) or in the absence of LPS, did not exhibit a remarkable difference in TREM2 protein levels with respect to those measured in untreated cells (Supplementary Figures S2 and S3). On the basis of our previous experiments showing that cell exposure to a 25 μM concentration of each polyphenol gave the best protective effects in all three tested conditions (Figure 1), we used each polyphenol at this concentration. Then, the starved cells, either pre-treated or not for 4 h with 25 μM of each polyphenol, were exposed to LPS. We found that LPS induced a decrease in TREM2 levels on the surface of BV2 cells (Figure 2A,B). However, the LPS-induced reduction of TREM2 expression was completely reversed in cells exposed to OleA or HT (Figure 2A,B).

In our LPS-exposed BV2 cells, we also investigated the levels of CD68, a common pan-macrofage marker expressed on resting microglia. We found that CD68 expression was greater in LPS-treated microglia than in untreated controls cells and that cell pretreatment with either polyphenol did not induce a significant reduction of this marker (Figure 2A,C).

Overall, our data in LPS-exposed cells relative to cell sufferance, ROS production, TREM2 and CD68 expression are in good agreement with each other and indicate that OleA and HT significantly alleviate some of the negative effects of LPS-induced inflammation by reducing TREM2 imbalance while being ineffective where CD68 expression is concerned.

### 2.3. OleA and HT Ameliorate the Imbalance of TREM2 Signaling

**Figure 3:**
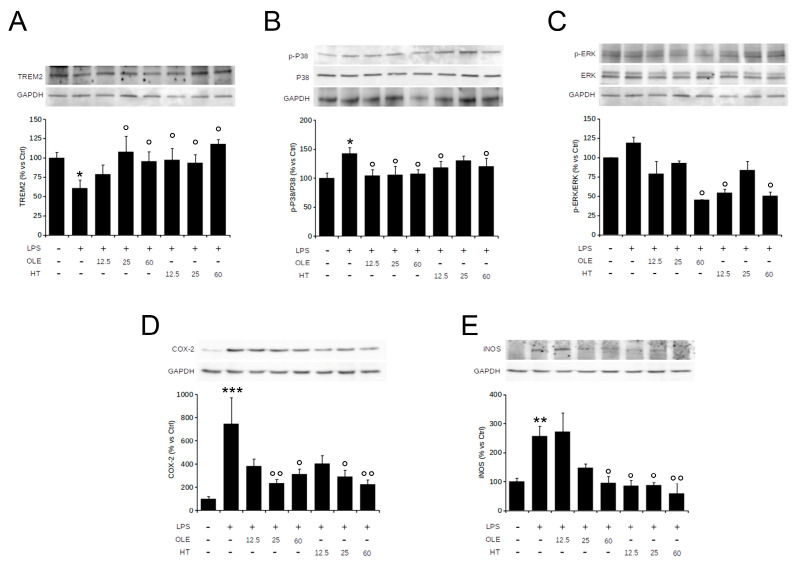
OleA and HT inhibit the pro-inflammatory TREM2-ERK-p38 pathway signaling and reduce the LPS-induced iNOS and COX-2 expression. BV2 cells were starved for 16 h and then treated for 4 h with different concentrations of OleA or HT (12.5, 25, 50 μM). Then, LPS (1.0 μg/mL) was added to the culture media for 24 h. Representative Western blots for TREM2 (**A**); p-p38 (**B**); p-ERK1/2 (**C**); COX-2 (**D**); iNOS (**E**) are shown. Signal quantification was obtained by densitometry analysis. Error bars represent the standard errors p-value: *: *p* < 0.05, **: *p* < 0.01 ***: *p* < 0.001 vs. the untreated cells, °: *p* < 0.05, °°: *p* < 0.01, vs. the LPS-stimulated cells. Tukey’s test, (*n* = 3).

Oxidative stress activates mitogen-activated protein kinases (MAPKs), including stress-activated p38 mitogen-activated protein kinase (p38 MAPK) and extracellular signal-regulated kinase (ERK) that, in turn, regulate a broad variety of physiological activities and exert important roles in the modulation of microglial activation and the inflammatory responses. Indeed, ERK1/2 and p38 MAPK phosphorylation induce the release of many pro-inflammatory molecules such as COX-2 and iNOS. On the basis of this background, we explored the effect of OleA and HT on LPS-induced cell damage by examining the activation of the MAPK pathway and mediator release by Western blotting. First, we confirmed previous data on the ability of our polyphenols to reduce LPS-induced TREM2 imbalance (Figure 3A); then, in LPS-stimulated BV2 cells, we found an increase of the levels of ERK (albeit not reaching a statistical significance) and p38 phosphorylation by 40% and 25%, respectively, as compared to controls. The increase in p38 and ERK phosphorylation was completely suppressed by cell pre-treatment with OleA (Figure 3B,C), whereas HT dramatically reduced ERK phosphorylation and, partially, that of p38 (Figure 3B,C). As shown in Figure 3D,E, OleA and HT significantly and dose-dependently reduced the iNOS and COX-2 expression induced by LPS. Notably, we observed an important anti-inflammatory effect resulting from cell treatment with HT even at the lowest concentration (12.5 μM), whereas in OleA-treated cells the maximum effect was achieved only at the highest doses (25 and 50 μM) of the polyphenol. Overall, these results indicate that each polyphenol is able to reduce the imbalance of TREM-2 and LPS-induced inflammatory reaction in BV2 cells through inhibiting the activation of the p38 and ERK signaling and downregulating the iNOS and COX-2 expression levels.

### 2.4. OleA and HT Suppress the LPS-Induced Phosphorylation and Nuclear Translocation of NF-κB

**Figure 4:**
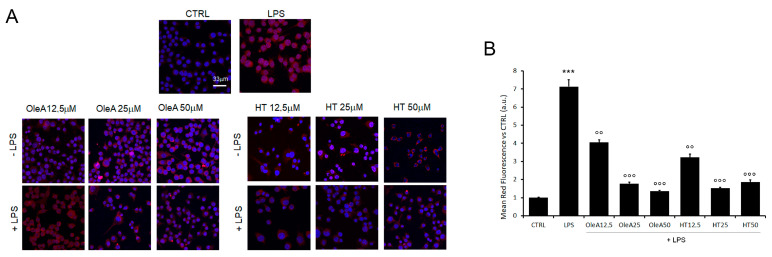
OleA and HT restrain NF-κB activation. (**A**) Representative confocal images of BV2 cells starved for 16 h, treated for 4 h with OleA or HT (12.5, 25 or 50 μM) and then exposed for 24 h to LPS (1.0 μg/mL) (+LPS). The cells were labelled with primary rabbit anti-pNF-κB antibody and with Alexa 568- conjugated secondary anti-rabbit antibody (red fluorescence). Cell nuclei were labelled with HOECHST dye (blue fluorescence). Scale bar 33 μm. (**B**) Semi-quantitative analysis of red fluorescence signals vs. untreated cells (CTRL) is shown. The analysis was performed by ImageJ software as the integrated intensity of at least 3 images for each of 3 independent experiments. Statistics: ***: *p* < 0.001 vs. CTRL; °°: *p* < 0.01; °°°: *p* < 0.001 vs. LPS.

NF-κB activation drives the transcription of many pro-inflammatory cytokines and, can negatively regulate TREM2 expression. It has also been suggested that brain-permeable inhibitors of NF-κB signaling can prevent or slow AD progression. Among these inhibitors, several plant polyphenols could play an important role due to their reported ability to interfere with the activation of NF-κB. Taking into consideration all these data, we evaluated the extent of the inflammatory response in BV2 cells treated with either EVOO polyphenol in terms of the activation state of NF-κB; in particular, we investigated the effect of cell pre-treatment with OleA or HT on NF-κB activation in terms of nuclear translocation of its phosphorylated form (pNF-κB). We focused on the early signaling events occurring within the first one hour. BV2 cells were kept under starvation conditions for 16 h, pre-treated for 4 h with OleA or HT (12.5 µM, 25 µM or 50 µM) and then exposed to LPS (1.0 µg/mL) for 45 min (Figure 4). The cells treated with either polyphenol in the absence of LPS did not exhibit any modification of NF-κB activation; the phosphorylated form of the latter was detected predominantly in the nucleus of LPS-activated BV2 cells, where it can regulate the transcription of genes involved in the inflammatory response. Finally, the cells pre-treated with OleA or HT displayed a dose-dependent reduction of nuclear translocation of pNF-κB. Overall, these results suggest that the anti-inflammatory action of EVOO polyphenols results from, or includes, the inhibition of NF-κB signaling (Figure 4).

### 2.5. Olive Polyphenols Decrease the LPS-Induced Production of Cytokine, Chemokine and Nitric Oxide

**Figure 5:**
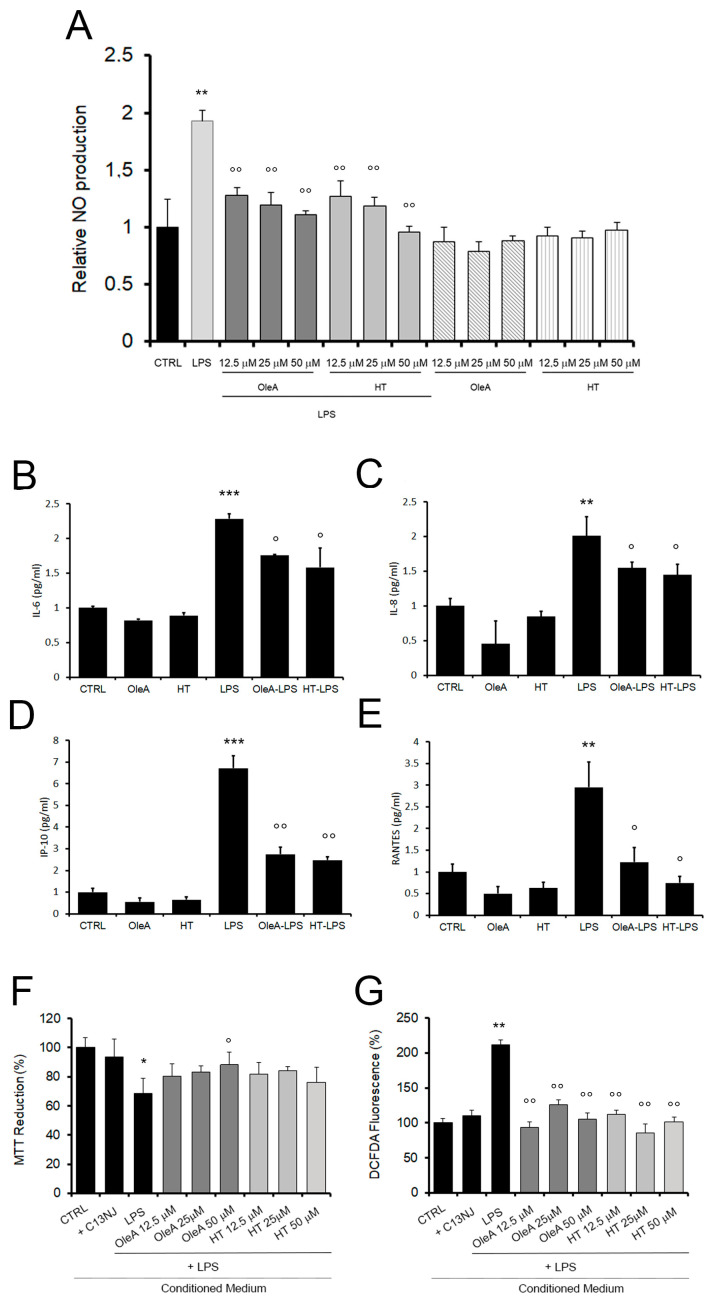
OleA and HT reduce the release of pro-inflammatory citokines and chemokines in LPS-exposed cells. (**A**) NO release, as assessed in BV2 cells pre-treated with different concentrations of OleA or HT without or with LPS (1.0 μg/mL). The data were reported as mean of triplicate analysis of three independent experiments ± SE. (**B**–**E**). The release of cytokines and chemokines was assessed by Bio-Plex analysis of human microglia cells, C13NJ. (**F**,**G**) Conditioned medium collected from C13NJ cells exposed to LPS with or without pre-treatment with either polyphenol induced a different cellular response in SH-SY5Y neuronal cells. Mitochondrial function and ROS generation in the latter cells were analysed by the MTT (**F**) and DCFDA fluorescence (**G**) assays. In these experiments, SH-SY5Y cells were exposed for 24 h to a conditioned medium obtained from: C13NJ cells (+C13NJ), C13NJ cells exposed for 24 h to LPS (LPS), C13NJ cells pre-treated for 4 h with either polyphenol and then exposed for a further 24 h to LPS. Statistics: *: *p* < 0.05; **: *p* < 0.01; ***: *p* < 0.001 vs. untreated cells (CTRL). °: *p* < 0.05; °°: *p* < 0.01; vs. LPS treated.

Once we had assessed the effect of OleA and HT on NF-κB signaling, we also investigated the relation, if any, between the two polyphenols and the production of nitric oxide (NO), another master regulator of inflammatory response. BV-2 cells showed a small, yet significant, increase of NO secretion in response to LPS treatment (Figure 5A), whereas no change in NO release was observed in cells pre-treated with either polyphenol before exposure to LPS (Figure 5A). Cytokine and chemokine levels were measured in a human microglia cell line, C13NJ (according to the assay manufacturer). We previously compared the behavior of these cells to that of BV2 following stimulation with either polyphenol or LPS. The results shown in the Supplementary Figure S4 indicate a similar response in the two cell lines and therefore, we performed the subsequent analysis only with the human cell line.

Cytokine and chemokine concentrations in the culture media were assessed by Bio-Plex analysis (Figure 5B–F). At 24 h, we are able to detect 20 out of the 27 checked cytokines and chemokines. Notably, the presence of either polyphenol alone did not affect the production of any of these molecules, except for three cases in which HT slightly increased the values of FGF, TNFα and VEGF with respect to the control cells (Table 1). In cells exposed for 24 h to LPS, 14 cytokines/chemokines were significantly increased (except PDGFbb, IL1ra, IL15, FGFbasic, MCP1) as compared to control cells, in accordance with the LPS pro-inflammatory activity. The LPS-induced increase of interleukin-6 (IL-6), interleukin-8 (IL-8), γ-interferon inducible protein-10 (IP-10) and regulated on activation, normal T-cell expressed and secreted (RANTES) and the production of MIP1b were significantly attenuated in cells pre-treated with either polyphenol. No decrease of LPS-induced expression of IL-1b, IL5, IFNγ, IL4, TNFα was observed in cells pre-treated with either polyphenol. In addition, only OleA reduced the LPS-induced expression of Eotaxin, and only HT reduced the LPS-induced expression level of GM-CSF.

The LPS-induced secretion of pro-inflammatory cytokines and potentially neurotoxic compounds also resulted in a significant sufferance of SH-SY5Y human neuroblastoma cells. Indeed, when the latter cells were exposed to a conditioned medium taken from microglial cells exposed to LPS, a high level of mitochondrial sufferance was observed, as assessed by the MTT (Figure 5F) and ROS production (Figure 5G) assays. Conversely, the neuronal cell viability was improved and ROS production was reduced in the presence of conditioned media collected from C13NJ cells pre-treated with either polyphenol before exposure to LPS. Taken together, these results confirm the anti-inflammatory effect of the two investigated EVOO polyphenols and their role in mitigating microglia-mediated neuroinflammatory response.

### 2.6. M2 Phenotype Characterization

**Figure 6:**
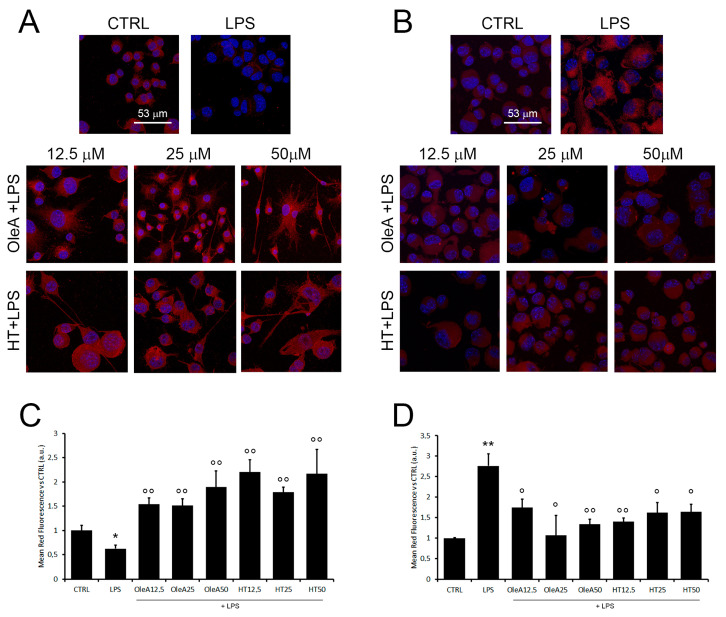
OleA and HT promote microglia polarization towards the M2 phenotype. Representative confocal images of BV2 cells starved for 16 h, treated for 4 h with OleA or HT (12.5, 25 and 50 μM) and then exposed for 24 h to LPS (1.0 μg/mL). The cell membranes were labelled with anti-CD163 (**A**) and anti-Iba1 (**B**) rabbit primary antibody and with secondary anti-rabbit-568-(conjugated antibody (red signals). The cells’ nuclei were labelled with HOECHST dye (blue signal). (**C**,**D**) Semi-quantitative analysis. Mean values of the mean red fluorescence signals ((**C**), CD163; (**D**), Iba1) vs. untreated cells (CTRL). Semi-quantitative analysis was performed by ImageJ software as integrated intensity of at least 3 images for each of 3 independent experiments. Statistics: *: *p* < 0.05; **: *p* < 0.01 vs. CTRL; °: *p* < 0.05; °°: *p* < 0.01 vs. LPS.

We also investigated, by confocal microscopy, the effect of OleA and HT on the BV2 cell phenotype resulting from microglia polarization under pro-inflammatory conditions by monitoring two markers of the M1 and M2 phenotype. In detail, we observed that LPS reduced the anti-inflammatory phagocytic surface markers CD163, scavenger receptor for the hemoglobin–haptoglobin complex, and upregulated the ionized calcium-binding adapter molecule 1, Iba1, considered a generic marker of microglia rather than a marker of an activated subset, although several studies suggest that Iba1 expression increases with microglial activation. These results are consistent with the effects of LPS in terms of activation of the pro-inflammatory phenotype of microglia. However, in contrast to the cell exposure to LPS, we found that cell pre-treatment with either polyphenol before exposure to LPS increased the signals of CD163 (Figure 6A), while it reduced Iba1 expression (Figure 6B). These data confirm the anti-inflammatory power of both polyphenols and also their ability to be triggers of the microglia polarization towards the anti-inflammatory, M2 phenotype.

## 3. Discussion

It is well known that aging-related neurodegeneration can also be caused by a chronic neuroinflammatory state, such as that reported in AD, where the microglia polarization towards a persistent pro-inflammatory phenotype plays a key pathogenetic role [37].

Therefore, the development of disease-modifying treatments targeting neuroinflammation may be an appropriate approach to address one of the main causes of the onset of AD and other neurodegenerative pathologies and to delay their progression.

To date, the absence of effective anti-inflammatory treatments for AD may be due to the lack of a correct and complete comprehension of the neuroinflammation process. Exhaustive clarification of the protective and pathogenic features of the inflammatory processes could uncover new targets for more efficacious treatment, leading to an improvement in AD-associated morbidity.

Today, alternative therapies have been promoted because of the long history of use in human health of medicinal plants. Indeed, natural compounds generally have low levels of side effects, good tolerability in patients and relative cost-effectiveness. The main polyphenols in EVOO, OleA and its main metabolite, HT, are well studied and can be easily administered through food or in other formulations and do not present adverse reactions in humans. In particular, OleA and HT exert their neuroprotective effects by their ability to cross the blood–brain barrier (BBB) [38,39,40]; indeed, the aglycone form can cross membranes by a passive diffusion [38]. This peculiarity makes EVOO polyphenols potential nutraceutical tools and promises the realistic use of these active principles in the treatment of neuroinflammation.

The goal of our research was to assess the effects of natural EVOO polyphenols in a state of BV-2 microglia cells activation induced by an acute stress, such as stimulation with LPS. LPS is a Gram-negative bacterial component that acts as a TLR4 ligand to arouse several downstream signals transduction, such as MAPK signaling and activation of NF-κB, which in turn, as a key transcription factor, increases the expression of pro-inflammatory molecules [41]. Herein, we demonstrated that OleA and HT are able to significantly prevent LPS-induced cytotoxicity in BV2 microglia cells, such as mitochondrial damage and increased ROS production within the cells. The anti-oxidant effects induced by OleA and HT are associated with a reduction in the nuclear translocation of NF-kB, pro-inflammatory agents, including COX-2, iNOS, NO and IL-6, IL-8, IP10 and RANTES, whose increased levels are correlated with an increased risk of dementia and neurological disorders [42,43,44]. Moreover, the attenuation of proinflammatory factors release by LPS-exposed microglia in the presence of polyphenols was also corroborated by the partial prevention of neuronal death observed in an in vitro model of neurotoxicity in which SH-SY5Y neurons were cultured in a conditioned medium from human microglia exposed to LPS with or without pre-treatment with polyphenols. Collectively, these results suggest that OleA and HT switch the polarization of LPS-activated BV2 cells from M1 to a mainly M2 phenotype, as indicated not only by the significant decrease in inflammatory cytokine production and neurotoxicity, but also by the prevention of changes in Iba-1 and CD163 levels caused by LPS treatment. CD163, a member of the group B scavenger receptor cysteine-rich (SRCR) family, has been considered a specific marker with homeostatic capacity and with strong anti-inflammatory and phagocytic properties in microglia [45]. Iba-1 protein expression, in particular, in combination with other markers such as iNOS, COX2, IL6, well mimics microglial activation [46,47]. Reactive microglia observed in aging and in neurodegeneration results in a loss of neuro-protective effects linked to the homeostatic phenotype, which normally assist neural repair and protect against neurodegenerative disorders by phagocytosing cellular debris and misfolded protein aggregates [37,48]. Consistent with maintaining the anti-inflammatory state induced by EVOO polyphenols, we found that these molecules contribute to maintaining microglia in a homeostatic phenotype by preventing LPS-induced TREM2 downregulation. Newly reported findings highlight the negative modulatory effect of TREM2 on the pathophysiology of neuroinflammation. TREM2 has been implicated in the attenuation of pro-inflammatory cytokines release, stimulation of phagocytosis, improvement of microglial proliferation and maintaining the expansion and survival of microglia by negatively modulating the TLR4-mediated activation of NF-κB signaling cascades [49,50]. In APP/PS1 mice, the downregulation of TREM2 mediated by TLR4, with increased release of proinflammatory cytokines, was correlated with cognitive dysfunction, Aβ accumulation and neuroinflammation, and it was associated with the activation of the ERK1/2 and MAPK signaling pathway [51].

Our data indicate that OleA and HT, by upregulating the expression of TREM2 and CD163 on microglia, and by decreasing Iba-1 levels and citockine release, prevent the MAPK-ERK1/2 pathway’s activity and suppresses cytokine production following the recognition of LPS by TLR4. Therefore, we may hypothesize that EVOO polyphenols may ameliorate the inflammatory environment resulting from microglial activation through enhancement of the phagocytotic and anti-inflammatory capacities of microglia related to an antagonistic mechanism to TLR4 [52].

It is unclear how OleA and HT modulate TREM2 and CD163 expression, for example, if they act in competition with LPS for TLR4 signaling pathways [53], as suggested also in Xu et al. and Zhand et al.’s papers [54,55], and further studies will be essential to understand the exact molecular mechanism of these EVOO polyphenols, also, for the possible design of new molecules with greater bioavailability and biological efficacy.

Since the reduction of neuroinflammation has been supposed an appealling strategy in the treatment of neurodegenerative disorders, these studies could open a new field of exploration on the potential neuroprotective actions of anti-inflammatory agents in neurodegenerative process occurring in the presence of reactive glia.

## 4. Materials and Methods

### 4.1. Cell Line and Culture Conditions

BV-2 microglia cells were obtained as previously reported, using an infection with the J2 virus [56]. Murine BV2 microglia were grown in RPMI supplemented with 2.0 mM L-glutamine, 100 μg/mL streptomycin, 100 U/mL penicillin and 10% FBS, at 37 °C in a 5.0% CO_2_ atmosphere. At 90% confluence, the cells were collected by scraping and seeded at the appropriate cell density. All subsequent experiments were performed in serum-free medium (starvation medium). The Human C13NJ microglia cells were prepared from embryos as described by Peudenier et al. [57]. The human microglial cell line C13NJ was maintained in DMEM supplemented with 10% FBS and 100 μg/mL streptomycin at 37 °C under 5.0% CO_2_. The microglia cell lines were kindly provided by Dr. Branca and maintained following his instructions [58]. The SH-SY5Y cell line is a thrice cloned subline of the neuroblastoma cell line SK-N-SH (ATCC HTB-11 Manassas, VA, USA); cells were cultured at 37 °C in complete medium (50% HAM, 50% DMEM, 10% fetal bovine serum, 3.0 mM glutamine, 100 U/mL penicillin and 100 μg/mL streptomycin) in a humidified, 5.0% CO_2_ incubator. All materials used for cell culture were from Merck–Sigma-Aldrich (Milano, Italy).

### 4.2. Preparation of Oleuropein Aglycone (OleA) and Hydroxytyrosol (HT) Samples

Oleuropein was purchased from Extrasynthese (Genay, France) and deglycosilated by treatment with almond β-glucosidase (EC 3.2.1.21, Sigma-Aldrich, St. Louis, Germany), as previously described [59]. Stocks of OleA (50 mM) were kept frozen and protected from light and were used within the same day once opened.

HT was purchased from Merck–Sigma-Aldrich (Milano, Italy). The powder was dissolved in an aqueous solution at 100 mM final concentration and stored at −20 °C, as previously reported [60].

### 4.3. MTT Reduction Assay

The microglia cells (BV2 and C13NJ) were grown in 96-well plates (1.0 × 10^4^ cells/well) for 24 h and pre-treated for 4 h with different concentrations of OleA or HT (12.5 μM, 25 μM, 50 μM, unless otherwise stated). Then, the cells were stimulated with LPS (1.0 μg/mL) for 24 h. We also assessed cell co-treatment with OleA or HT and LPS for 24 h. After cell treatments, 100 μL of MTT solution (0.5 mg/mL) were added to each well and the cells were maintained in the dark at 37 °C for 1 h. After washing out the supernatant, the insoluble formazan product was dissolved in 80 μL/well of dimethyl sulfoxide (DMSO). The absorbance was measured at 595 nm using the iMARK microplate reader (Bio-Rad, Segrate (MI), Italy). Data were expressed in terms of a percentage with respect to untreated control cells.

### 4.4. Intracellular ROS Determination

BV2 and C13NJ microglia cells were seeded in a 96-well plate at a density of 1 × 10^4^ cells/well for 24 h. After appropriate cell treatments, the ROS-sensitive fluorescent probe DCFH-DA (10 μM) was added to each well and incubated in the dark for 1.5 h at 37 °C to detect intracellular ROS levels. The DCF fluorescence intensity was detected at an excitation/emission wavelength of 485/538 nm, respectively, using a fluorescence microplate reader (Fluoroskan AscentTM FL Microplate Fluorometer, Thermo Fisher Scientific, Waltham, MA, USA). Data were expressed in terms of a percentage with respect to untreated control cells and normalized with respect to the corresponding MTT data.

### 4.5. Western Blot Analysis

BV2 (5 × 10^5^ cells/well) were grown in a 6-well plate for 24 h, then subjected to the different treatments and eventually washed with cold PBS and lysed in 80 μL of Laemmli buffer (62.5 mM Tris-HCl, pH 6.8, 10% (*w*/*v*) SDS, 25% (*w*/*v*) glycerol) without bromophenol blue. The whole cell lysates were collected and boiled at 95 °C for 10 min and then centrifuged at 12,000× *g* for 10 min at 4 °C. Total protein concentration was measured by the BCA method. Twenty-five µg of total proteins were loaded in precast SDS-PAGE gels (Biorad, Segrate (MI), Italy) and then transferred onto a PVDF membrane by a Trans-Blot Turbo Transfer Pack (Biorad). The immunoblots were incubated at R.T. in PBS containing 5.0% (*w*/*v*) BSA, 0.1% (*v*/*v*) Tween 20, and probed with the appropriate primary and secondary antibodies. The latter were specific for TREM2 (Merck–Sigma-Aldrich, Rabbit), phospho-P38 (Santa-Cruz Biotechnology, Dallas, TX, USA, mouse), P38α (Santa-Cruz Biotechnology, Dallas, TX, USA, mouse), p-ERK (Cell Signaling, Danvers, MA, USA, rabbit), ERK (Cell Signaling, Danvers, MA, USA, mouse), GAPDH (Cell Signaling, Danvers, MA, USA, mouse), COX-2 (Cell Signaling, Danvers, MA, USA, rabbit), iNOS (Cell Signaling, Danvers, MA, USA, rabbit). At the end of the incubation, the membranes were repeatedly washed in 0.5% (*v*/*v*) PBS-Tween^®®^-20 solution and the protein bands were detected using the Clarity Western ECL solution. Chemiluminescent signals were acquired by using the AmershamTM 600 Imager imaging system (GE Healthcare Life Science, Pittsburgh, PA, USA); the densitometric analysis was carried out using the Quantity One software (4.6.6 version, Bio-Rad, Segrate (MI), Italy).

### 4.6. Immunofluorescence

Subconfluent BV2 cells grown on glass coverslips (5 × 10^4^ cells/well) in a 24 well-plate were exposed for 4 h to different concentrations of polyphenols and then treated for 24 h with LPS. Cell nuclei were labelled for 30 min at room temperature with 1.0 µg/mL Hoechst 33342. Then, the cells were fixed in 2.0% buffered paraformaldehyde for 6 min and permeabilized by treatment with a 1:1 acetone/ethanol solution for 4.0 min at room temperature, washed with PBS and blocked with PBS containing 0.5% BSA and 0.2% gelatin. The antibodies used for immunofluorescence were specific for TREM2 (1:500; Merck–Sigma-Aldrich, rabbit), CD68 (1:300, Santa-Cruz Biotechnology, Dallas, TX, USA, mouse), p-NF-kB p65 (1:500, Cell-Signaling, Danvers, MA, USA, Rabbit), and CD163 (1:500, GeneTex, Irvine, CA, USA Mouse). After incubation for 1 h at room temperature with primary antibody diluted in blocking solution, the cells were washed with PBS for 30 min under stirring and then incubated with Alexa568-conjugated anti-rabbit secondary antibody (Molecular Probes, Eugene, OR, USA) or Alexa 488-conjugated anti-mouse antibody diluted 1:100 or 1:200 in PBS, respectively. Finally, the cells were washed twice in PBS and once in distilled water to remove non-specifically bound antibodies. Multicolor images were collected using a Leica TCS SP8 scanning microscope (Leica, Mannheim, Germany) equipped with 63×, 1.4–0.6 NA, oil, HCX Plan APO lens. The images were captured using the Leica LAS-AF image acquisition software (Versions 5.1.0). Photo montages and signals quantification were generated using the FiJi software (Version 8).

### 4.7. NO Assay

The cells were pleated at the density of 7 × 10^4^ cells/well in a 12 well-plate. Supernatants collected from BV2 cells treated with different conditions were evaluated for nitric oxide (NO) production according to the Griess reaction previously reported [61]. For each treatment, 50 µL of cell culture medium were collected and mixed with equal volumes of Griess reagent before incubation for 15 min at room temperature. As a reference, sodium nitrite in the 0–50 µM range was used to determine nitrite concentration in the sample. Absorption intensity was measured at the wavelength of 540 nm using a microplate reader. Data were normalized to cell viability and expressed in terms of a percentage with respect to untreated control cells. Treatments were completed at least three times and data are expressed as mean ± SEM.

### 4.8. Determination of Cytokine Release

C13NJ cells were grown (5 × 10^5^ cells/well) in 6 well-plates, exposed for 4 h to different concentrations of polyphenols and then treated with LPS for 24 h. After treatments, the medium was collected, centrifuged at 14,000× *g* to remove debris, and then stored as aliquots at −80 °C. Conditioned media were screened for the concentration of interleukin (IL)-1ra, IL-1b, IL-2, IL-4, IL-5, IL-6, IL-7, IL-8, IL-9, IL-10, IL-12 (p70), IL-13, IL-15, IL-17A, basic fibroblast growth factor (FGF), eotaxin, granulocyte-colony stimulating factor (G-CSF), granulocyte macrophage-colony stimulating factor (GM-CSF), interferon-γ (IFN-γ), interferon-γ inducible protein 10 (IP-10), monocyte chemoattractant protein-1 (MCP1), macrophage inflammatory protein-1 (MIP-1) α, MIP-1β, C–C motif chemokine ligand 5 (CCL5)/RANTES, TNF-α, platelet derived growth factor (PDGF-BB) and vascular endothelial growth factor (VEGF) using the Bio-Plex Pro Human Cytokine Grp I Panel 27-Plex kit (cat. no. M500KCAF0Y, Bio-Rad, Segrate (MI), Italy) according to the supplier’s instructions. The magnetic bead-based assay was performed on a Bio-Plex 200 analyzer with Bio-Rad Bio-Plex Manager (Bio-Rad, Hercules, CA, USA) [62].

### 4.9. Assay of Cytotoxicity to Neuronal Cells of the Factors Released by Microglia

C13NJ microglia cells were grown in 96-well plates (1 × 10^4^ cells/well) for 24 h and treated under starvation conditions with either polyphenol at different concentrations for 4 h and then with LPS (1.0 μg/mL) for 24 h. The SH-SY5Y cells were seeded into 96-well plates at a density of 6 × 10^3^ cells/well in fresh complete medium and grown for 24 h. Then, the cells were treated with the conditioned medium collected from the LPS-treated C13NJ cells. After 24 h of cell treatment with the conditioned medium, the MTT and ROS assays were performed in accordance with the protocol reported in the Section 4.

### 4.10. Data Analysis

Data are reported as mean ± standard error of triplicate values collected in at least three independent experiments. Unless otherwise specified, the statistical analysis of the data was performed using the one-way analysis of variance (ANOVA) and pairwise comparisons were performed using Tukey HSD method. The Western blotting statistical analysis was performed by the Kruskal–Wallis test followed by the Conover post hoc test.

## 5. Conclusions

In conclusion, our study reports for the first time that the anti-inflammatory effects of OleA and HT proceed through TREM2 upregulation, which favors M2 polarization. These data provide novel insights into the role played by the main EVOO polyphenols in modulating microglial differentiation and modifying its polarization. These new abilities we described of the main EVOO polyphenols could be used to enhance protective microglial activity and may be adopted as a therapeutic (or preventive) approach or in addition to conventional therapy to enable resident microglia to trigger an early response against neurodegenerative disorders.

## Figures and Tables

**Table 1:** Panel of cytokines and chemokines analyzed. Mean reported vs. CTRL and standard error appertaining to the 27 detectable cytokines and chemokines were quantified using the Bio-Plex multiplex beads system.

pg/mL	vs. CTRL					*p* Value vs. CTRL			*p* Value vs. LPS	
	LPS	OleA	HT	OleA-LPS	HT-LPS	LPS	OleA	HT	OleA-LPS	HT-LPS
PDGF-bb	1.0903 ± 0.09	0.6379 ± 0.168	0.7284 ± 0.09	1.2715 ± 0.09	1 ± 0.12	0.21	0.052	0.047		0.21
IL-1b	2.1052 ± 0.10	1.3421 ± 0.1438	1.2105 ± 0.1053	1.5526 ± 0.4038	1.5 ± 0.3163	0.0042	0.040	0.036	0.22	0.36
IL-1ra	1.1088 ± 0.0982	0.4309 ± 0.5	0.5899± 0.2696	1 ± 0.1	1.0560 ± 0.053	0.21		0.061	0.21	0.35
IL-2	OOR<	OOR<	OOR<	OOR<	OOR<					
IL-4	1.5913 ± 0.1613	0.5484 ± 0.2391	0.9139 ± 0.1811	1.3118 ±0.1613	1.5806 ± 0.1839	0.035	0.069	0.33		0.47
IL-5	1.2829 ± 0.0368	0.7541 ± 0.0262	0.8543 ± 0.054	1.1453 ± 0.0393	1.1514 ± 0.1415	0.004	0.018	0.046	0.027	0.24
IL-6	2.2838 ± 0.0731	0.8197 ± 0.0167	0.8890 ± 0.0409	1.7589 ± 0.0134	1.5844 ± 0.2757	0.0008	0.11	0.047	0.042	0.013
IL-7	OOR<	OOR<	OOR<	OOR<	OOR<					
IL-8	2.1090 ± 0.2778	0.4524 ± 0.3283	0.8465 ± 0.077	1.5480 ± 0.0831	1.4489 ± 0.3538	0.0033	0.0539	0.092	0.024	0.026
IL-9	1.3405 ± 0.0719	0.6024 ± 0.1038	0.8301 ± 0.0639	1.0610 ± 0.061	1.0457 ± 0.1105	0.033	0.18	0.057	0.023	0.15
IL-10	OOR<	OOR<	OOR<	OOR<	OOR<					
IL-12(p70)	OOR<	OOR<	OOR<	OOR<	OOR<					
IL-13	OOR<	OOR<	OOR<	OOR<	OOR<					
IL-15	0.8931 ± 0.13	0.3265 ± 0.5023	0.7995 ± 0.1969	0.6237 ± 0.2631	1.0892 ± 0.2832	0.23		0.16	0.14	0.30
IL-17	OOR<	OOR<	OOR<	OOR<	OOR<					
Eotaxin	1.2051 ± 0.1702	0.5448 ± 0.3412	0.9102 ± 0.0986	0.8653 ± 0.0519	1.1666 ± 0.1429	0.211	0.067	0.21	0.12	0.44
FGF basic	1.0424 ± 0.3307	0.2324 ± 0.1673	1.3788 ± 0.1958	0.8969 ± 0.1562	1.9322 ± 0.2904	0.45	0.19	0.11	0.34	0.12
G-CSF	1.4032 ± 0.2475	0.6091 ± 0.2208	0.6871 ± 0.0709	1.2981 ± 0.0703	0.6234 ± 0.4963	0.17	0.057	0.023	0.46	0.08
GM-CSF	3.6612 ± 0.3376	1.0414 ± 0.1134	0.6312 ± 0.1391	3.5369 ± 0.1074	1.0522 ± 0.47	0.07	0.37	0.045	0.26	0.14
IFN-γ	1.2878 ± 0.0959	0.5227 ± 0.4842	0.6060 ± 0.0758	1.0227 ± 0.3418	1.1439 ± 0.0964	0.057	0.10	0.017	0.39	0.11
IP-10	6.7186 ± 0.6911	0.5578 ± 0.3781	0.6473 ± 0.1995	2.7464± 0.3379	2.4714 ± 0.4727	0.00016	0.12	0.10	0.0023	0.0022
MCP-1 (MCAF)	1.1680 ± 0.4202	0.1596 ± 0.4202	0.5798 ± 0.5942	0.5966 ± 0.844	1.2941 ± 0.4313	0.36	0.091	0.21	0.16	0.21
MIP-1a	1.4 ± 0.233	0.85 ± 0.522	0.625 ± 0.39	1 ±0.212	0.4 ± 0.15	0.15		0.14	0.15	0.028
MIP-1b	1.6257 ± 0.130	0.7449 ± 0.122	0.8499 ± 0.048	1.3832 ± 0.093	1.3519 ± 0.144	0.048	0.054	0.029	0.21	0.22
RANTES	2.9518 ± 0.58	0.4961 ± 0.17	0.6270 ± 0.138	1.2278 ± 0.3339	0.7438 ± 0.150	0.0018	0.03	0.051	0.021	0.015
TNF-α	1.4048 ± 0.190	0.5788 ± 0.307	1.1433 ± 0.098	1.3056 ± 0.083	1.2037 ± 0.130	0.12	0.078	0.14	0.36	0.26
VEGF	1.4359 ± 0.091	OOR<	1.6765 ± 0.092	1.2007 ± 0.377	1.6316 ± 0.441	0.014		0.072	0.32	0.40

## Data Availability

Data are contained within the article and the Supplementary Materials.

## Supplementary Materials

The following supporting information can be downloaded at: https://www.mdpi.com/article/10.3390/ph16070933/s1, Figure S1: Effects of LPS or polyphenols on mitochondrial functionality and ROS production in BV2 cells; Figure S2: TREM2 expression levels in BV2 cells; Figure S3: TREM2 expression levels on microglia cell membrane after polyphenols treatment; Figure S4: Protective effects of OleA and HT on C13NJ exposed to LPS.
