# Supplementary material for: EVOO Polyphenols Exert Anti-Inflammatory Effects on the Microglia Cell through TREM2 Signaling Pathway
Source: Pharmaceuticals (Basel). 2023 Jun 27;16(7):933. doi: 10.3390/ph16070933 (PMC10384320; doi:10.3390/ph16070933)
Supplement: Supplementary file 1 [file pharmaceuticals-16-00933-s001.zip › pharmaceuticals-2417898-supplementary.pdf]

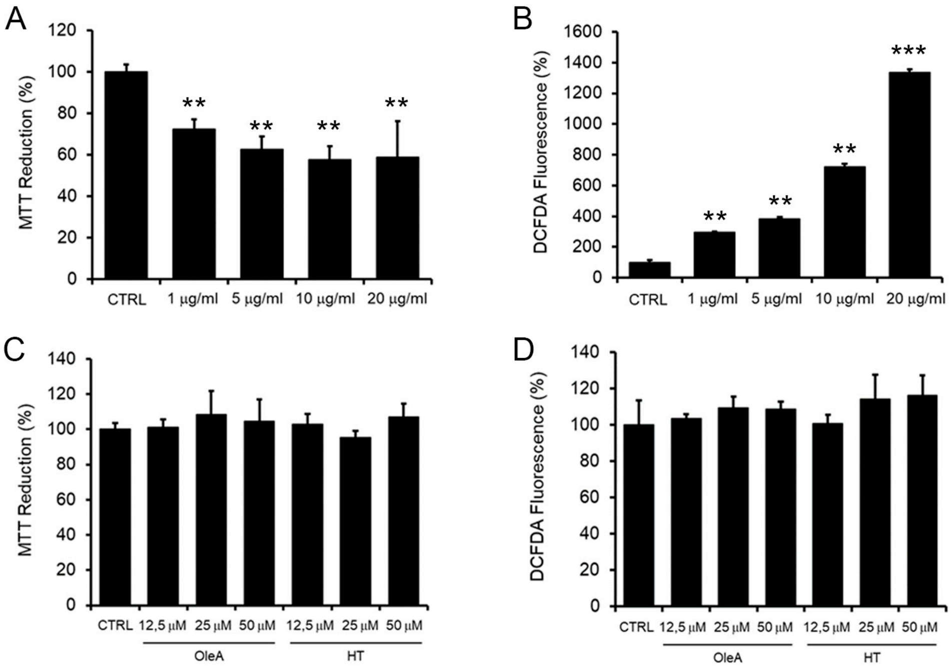

Figure S1. Effects of LPS or polyphenols on mitochondrial functionality and ROS production in BV2 cells. BV2 cells were starved for 16 h and treated for further 24 h with LPS (1, 5, 10 and 20 µg/mL) (**A, B**), OleA and HT (12.5, 25 and 50 µM) (**C, D**). The effects of the treatments on BV2 cells were assessed in terms of MTT reduction (**A, C**) and ROS production (**B, D**). The data were reported as mean of triplicate analysis of three independent experiment ± SE. Statistics: **: $p < 0{,}01$; ***: $p < 0{,}001$ vs untreated cells (CTRL).

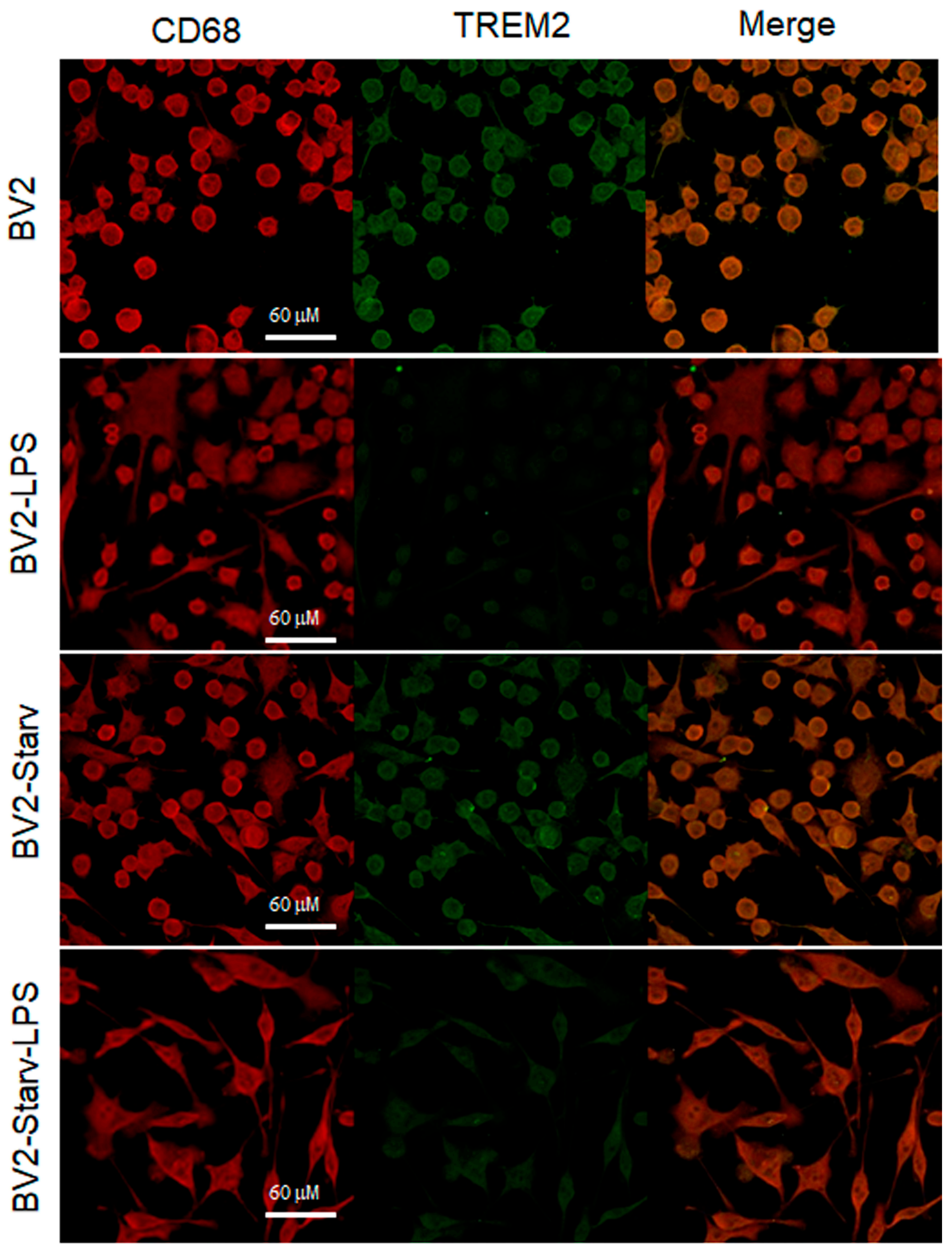

Figure S2. TREM2 expression levels in BV2 cells. BV2 cells were starved or not for 16 h and then treated with LPS for 24 h. The cell membranes were labelled with mouse primary antibody anti-CD68 and secondary anti-mouse 488-conjugated antibody (red signals). The receptor was labelled with the specific primary anti-TREM2 rabbit antibody and the secondary anti-rabbit Alexa 568-conjugated antibody (green signals). The two channels are shown both separated and in the merge mode.

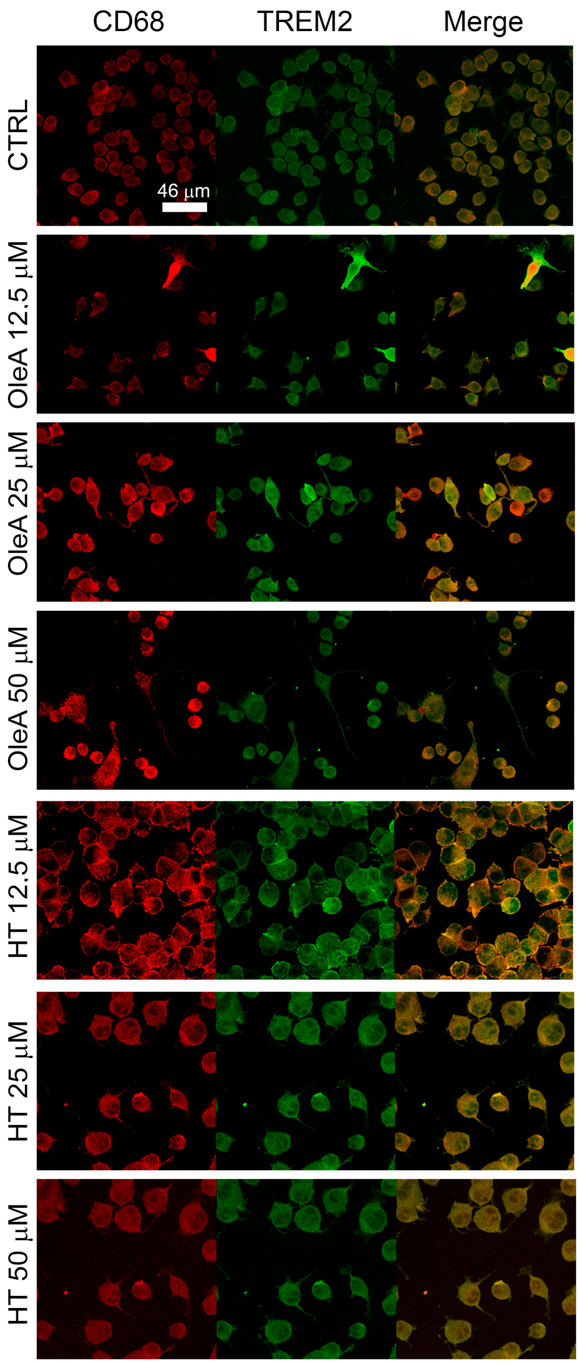

Figure S3. TREM2 expression levels on microglia cell membrane after polyphenols treatment. BV2 cells were starved for 16 h and then treated with different concentrations of OleA or HT (12.5, 25, 50 µM) for 24 h. The cell membranes were labelled with mouse anti-CD68 primary antibody and with anti-mouse 488-conjugated secondary antibody (red signals). The receptor was labelled with the specific primary rabbit anti-TREM2 antibody and the secondary anti-rabbit Alexa 568-conjugated antibody (green signals). The two channels are shown both separated and in the merge mode.

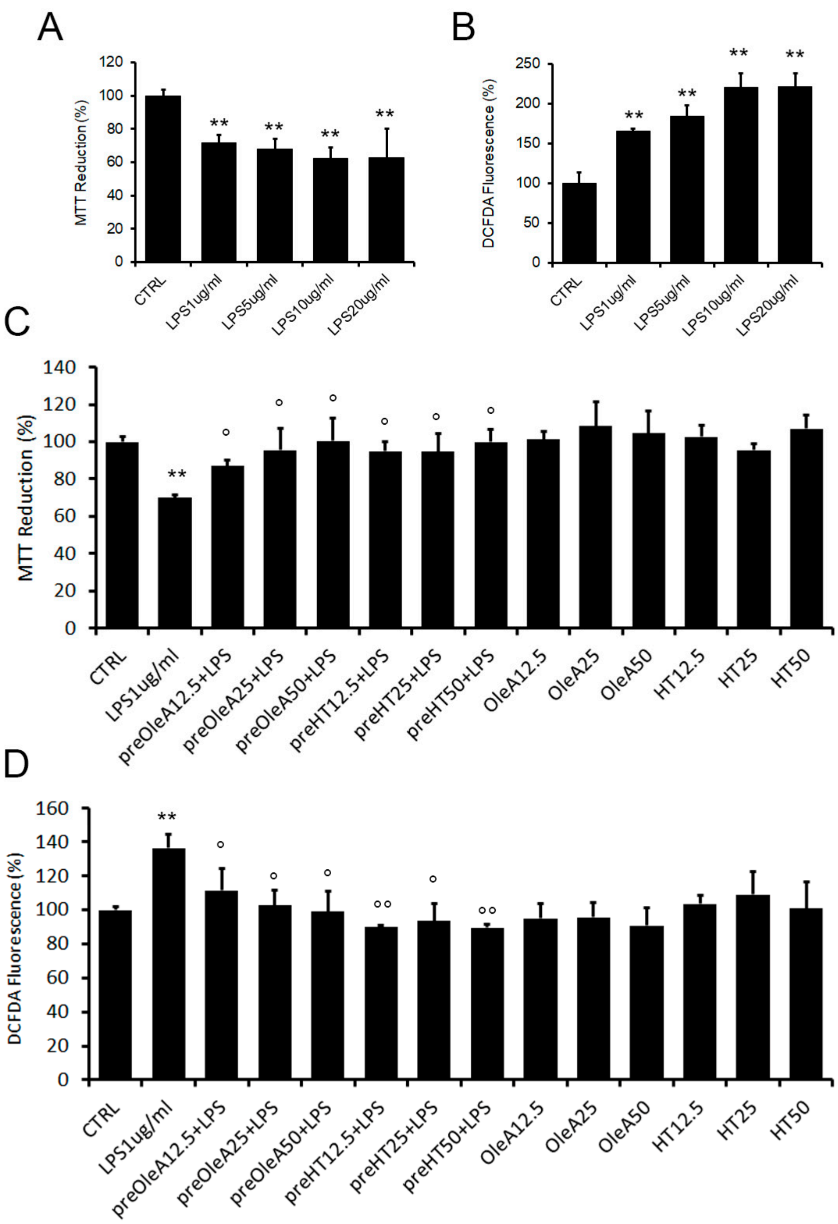

Figure S4. Protective effects of OleA and HT on C13NJ exposed to LPS. C13NJ cells were starved for 16 h and then treated for further 24 h with LPS (1, 5, 10 and 20 µg/ml). The effects of the treatments on the cells were assessed in terms of MTT reduction (A) and ROS production (B). The cells were starved for 16 h, treated with different concentrations of OleA or HT (12.5, 25, 50 µM) for 4 h (C, D) and then exposed to LPS (1.0 µg/µL) for 24 h. (C) Cytotoxicity of cell treatments, as assessed by the MTT assay. (**D**) ROS levels as measured by the DCFDA probe. The data were reported as mean of triplicate analysis of three independent experiment ± SE. Statistics: **: $p < 0,01$ vs untreated cells (CTRL). °: $p < 0,05$; °°: $p < 0,01$ vs LPS treated.
